# Transcutaneous auricular vagus nerve stimulation for long-term post-stroke cognitive impairment: a DTI case report

**DOI:** 10.3389/fnhum.2024.1473535

**Published:** 2024-10-08

**Authors:** Xixi Chen, Zhiqing Zhou, Kayee Chong, Jingjun Zhao, Yuwei Wu, Meng Ren, Yu Huang, Songmei Chen, Chunlei Shan

**Affiliations:** ^1^School of Rehabilitation Science, Shanghai University of Traditional Chinese Medicine, Shanghai, China; ^2^Department of Rehabilitation Medicine, Yueyang Hospital of Integrated Traditional Chinese and Western Medicine, Shanghai University of Traditional Chinese Medicine, Shanghai, China; ^3^Engineering Research Center of Traditional Chinese Medicine Intelligent Rehabilitation, Ministry of Education, Shanghai, China; ^4^Department of Rehabilitation Medicine, Tongren Hospital, Shanghai Jiao Tong University School of Medicine, Shanghai, China; ^5^Peking University People's Hospital, Beijing, China; ^6^Department of Rehabilitation Medicine, Shanghai No. 3 Rehabilitation Hospital, Shanghai, China; ^7^Yuanshen Rehabilitation Institute, Shanghai Jiao Tong University School of Medicine, Shanghai, China

**Keywords:** long-term post-stroke cognitive impairment, home-based, transcutaneous auricular vagus nerve stimulation (taVNS), diffusion tensor imaging (DTI), fractional anisotropy (FA)

## Abstract

**Purpose:**

Long-term post-stroke cognitive impairment (PSCI) exhibits an accelerated rate of long-term cognitive decline, which can impair communication, limit social engagement, and increase rate of institutional dependence. The aim of this case report is to provide evidence for the potential of home-based transcutaneous auricular vagus nerve stimulation (taVNS) for home-bound patients with severe, long-term PSCI.

**Methods:**

A 71-year-old male suffered a stroke two and a half years ago, which imaging reported foci of cerebral infarction visible in the left temporal and parietal lobes. The patient was performed taVNS twice a day for 30 min, 5 times a week for 8 weeks. The patient was evaluated the changes of cognitive function and brain white matter at 4 time points: baseline (t0), 4 weeks without taVNS after baseline (t1), 4 weeks of intervention (t2), and 8 weeks of intervention (t3). The effect of taVNS on white matter changes was visualized by DTI.

**Results:**

After 8 weeks of taVNS treatment, the scores of Montreal cognitive assessment improved and the time to complete the shape trails test decreased. The DTI results showed that white matter in bilateral dorsal lateral prefrontal cortex remodeled after taVNS.

**Conclusion:**

Eight-week home-based taVNS may be beneficial to long-term PSCI. Further studies of home-based taVNS treating patients with long-term PSCI are needed.

## Introduction

Post-stroke cognitive impairment (PSCI) is a common functional impairment after stroke, and has been defined as all problems in cognitive function that occur following a stroke, irrespective of the etiology (Quinn et al., 2021), typically impairing executive, memory, and visuoconstructional functions (Jokinen et al., 2015). The prevalence was ranging from 20 to 80% (He et al., 2023; Sun et al., 2014). Although cognitive function may initially exhibit signs of improvement and stabilization following stroke onset in patients with PSCI (Elgh and Hu, 2019), some individuals may subsequently experience long-term cognitive decline (Delavaran et al., 2017; Mahon et al., 2017). A study indicated that certain stroke patients may experience cognitive impairment for up to a decade. Compared to both non-stroke individuals and post-stroke patients without cognitive impairment, patients with PSCI had a faster rate of cognitive deterioration (Delavaran et al., 2017). There are many home-bound patients with chronic stroke who have developed and experienced cognitive decline. Cognitive impairment significantly affects patients’ quality of life, hampers communication, limits social engagement, and imposes a substantial economic burden on both families and society (Wagle et al., 2011; Zhang J. et al., 2021). Moreover, PSCI is associated with an elevated risk of mortality, dependence, and institutionalization, resulting in a significant social and economic burden, especially in cases of long-term PSCI with cognitive deterioration (Obaid et al., 2020; Chen et al., 2023). At present, there is insufficient evidence for drug treatment for PSCI. In the prevention of PSCI, recommended medications include antihypertensive, lipid-lowering, blood glucose control and other related drugs to control cerebrovascular disease risk factors. Non-pharmacological treatments such as compensation strategy training and direct cognitive skill training require high concentration, and patients’ compliance is poor. Non-invasive brain stimulation such as transcranial magnetic stimulation and transcranial electrical stimulation are relatively easy for patients to cooperate with, however, these devices need to be performed by a professional doctors and patients need to visit a hospital for treatment (Eskes et al., 2015; Quinn et al., 2021; Uzuner and Uzuner, 2023). Therefore, focusing on rehabilitation interventions of patients with long-term PSCI is crucial, especially those that allow patients to remain homebound.

Transcutaneous auricular vagus nerve stimulation (taVNS) is a non-invasive method targeting the auricular branches of the vagus nerve to modulate brain function. Existing research has highlighted the significant ameliorative impact of taVNS on central nervous system diseases (Choi et al., 2022; Murphy et al., 2023). The taVNS is neuroprotective against central nervous system injury by upregulating peroxisome proliferator-activated receptor *γ* expression (Eren and Yilmaz, 2022; Li et al., 2020). It was demonstrated that taVNS inhibited the expression of interleukin (IL)-1β, IL-6, and tumor necrosis factor-*α* (TNF-α), and promoted the functional recovery of cerebral ischemia/reperfusion-injured rats (Dash et al., 2022; Zhao et al., 2022). Notably, one study suggested that taVNS could increase the complexity of white matter microstructure (Jenkins et al., 2023). It has been shown that taVNS may inhibit post-stoke inflammation response in the white matter of cerebral ischemia model rats through TLR4/NF-κB and MAPK/NF-κB signaling pathways (Long et al., 2022; Xu et al., 2023). The taVNS can affect brain regions related to cognitive function, such as frontal cortex and hippocampus, with mediation through the nucleus tractus solitarius (Kraus et al., 2007; Yakunina et al., 2017). It was shown that taVNS promoted cerebrospinal fluid circulation in transient bilateral common carotid artery occlusion model mice, significantly improved discrimination index scores in the novel object recognition test rates of spontaneous alternations in the Y-maze test, resulting in the recovery of impaired cognitive function (Choi et al., 2022). Besides, taVNS can also modulates disrupted brain functional connectivity (Fang et al., 2016; Liu et al., 2020). Zhang found that taVNS was effective in reducing scores on Pittsburgh sleep quality index (PSQI) and reducing functional connectivity (FC) within the default mode network (DMN), FC between DMN and salience network and FC between DMN and the occipital cortex (Zhang S. et al., 2021). In addition, taVNS can improve mood problems. It can improve anxiety symptoms and modulate activation of the left triangle part of the inferior frontal gyrus in patients with Parkinson’s disease (Zhang et al., 2024). The taVNS instantly modulated the activity of the DMN and the cognitive control network in patients with major depression disorder and induced at least 12 weeks of clinical improvement (Rong et al., 2016; Sun et al., 2023). Together with its attributes of safety and portability, taVNS may be a potential option for long-term PSCI patients. Continuous supervised specific cognitive training and specialized equipment are not easy and convenient for home-bound PSCI patients. Thus, portable as well as easy-to-operate taVNS might be an effective strategy for home-based cognitive rehabilitation.

Diffusion tensor imaging (DTI) can be applied to white matter fiber tracking in the brain and quantitatively measure the integrity of white matter (Le Bihan et al., 2001). He et al. found that extensive damaged white matter microstructure in subacute PSCI patients (He et al., 2022). On voxel-wise analyses, reduced fractional anisotropy (FA) in almost all white matter tracts in early cognitive impairment patients with minor stroke (Zamboni et al., 2017). In this study, we selected region of interest (ROI) for the initial exploration of taVNS action on white matter in PSCI patients. The dorsolateral prefrontal cortex (DLPFC), cingulate gyrus, hippocampus and thalamus are thought to be closely related to cognitive function. The DLPFC impairment will lead to a series of impairments related to executive function and attention (Barbey et al., 2013a,b; Forbes et al., 2014). Anterior cingulate cortex (ACC) is involved in learning and outcome monitoring (Aly-Mahmoud et al., 2017; Kawai et al., 2015). The posterior cingulate cortex (PCC) is involved in maintaining attention and detecting environmental changes in order to change behavior (Leech and Sharp, 2014). The hippocampus regulates memory and plays an important role in spatial processing (Bird and Burgess, 2008). The thalamus plays integrative roles in cognition, ranging from learning and memory to flexible adaption (Wolff and Vann, 2018). The importance of these brain regions to cognitive function led us in this study to observe changes in their associated white matter after taVNS. Here, we provide evidence for the potential of home-based treatment for long-term PSCI patient and present the remodeling effects of taVNS on cognitively relevant white matter tracts by DTI.

## Materials and methods

The patient has given written consent for the publication of this case report. This case report was approved by the ethics committee of Shanghai No. 3 Rehabilitation Hospital (ID: SH3RH-2023-EC-022).

### The patient

A 71-year-old male suffered a stroke two and a half years ago, which imaging reported foci of cerebral infarction visible in the left temporal and parietal lobes. After early speech therapy, language skills improved and he was able to carry on a conversation, but after 6 months he developed mild cognitive deficits and had increasing problems with attention. Therefore, we used Non-language-based Cognitive Assessment to screen the patient. The patient scored 54 on a scale of 80, with a score of 70 or less indicating cognitive impairment. However, the patient subsequently returned home for personal reasons and stopped treatment. One year later, he came to us for cognitive treatment. As we were still able to communicate with the patient at this time, we chose to use the more sensitive and comprehensive Montreal cognitive assessment (MoCA) and mini-mental state examination (MMSE) for follow-up in order to more accurately assess his cognitive status. Given the patients’ satisfactory motor function and was not limited in activities of daily living, he opted to continue residing at home rather than rehospitalization post-discharge. To address the cognitive impairment, we initiated taVNS as a home-based treatment, assessing cognitive changes using specific cognitive function scales and monitoring the treatment’s mechanisms through DTI.

### The experimental protocol

**Figure 1:**
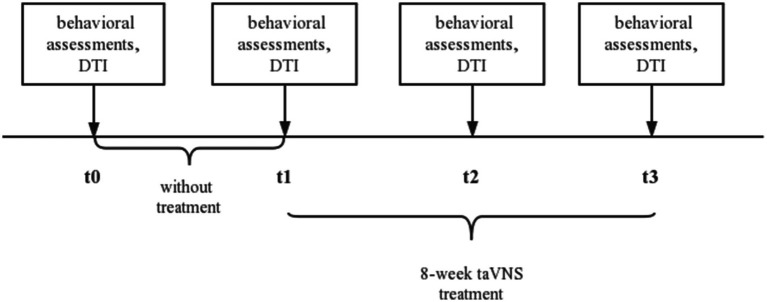
The experimental protocol. t0, baseline; t1, without treatment for four weeks; t2, 4-week taVNS treatment after t1; t3, 8-week taVNS treatment after t1. taVNS, transcutaneous auricular vagus nerve stimulation; DTI, diffusion tensor imaging.

We identified that patient could complete taVNS treatment with the help of family member, so we trained the family on taVNS operation in the hospital. We provided taVNS device for them to take home from the hospital and requested the family member to report patient daily treatment status to us via video twice per day. The patient received no other cognitive therapy. We ask the patient’s family to report the treatment status of the day daily. After we evaluated the patient at baseline (t0) and acquired the DTI, the patient was allowed to perform normal activities of daily living at home without treatment for four weeks. Then the patient would receive a second evaluation (t1) and DTI and began an 8-week treatment. We evaluated patient at two times: 4 weeks of intervention (t2) and 8 weeks of intervention (t3). We perform behavioral assessments and collect DTI data from patient before and after each session. Behavioral assessments included MoCA and MMSE for global cognitive function, auditory verbal learning test-HuaShan version (AVLT) for memory function, shape trails test (STT) for attention and executive function, Hamilton depression scale (HAMD) for depression symptom, Hamilton anxiety scale (HAMA) for anxiety symptoms, and PSQI for sleep. The experimental design is shown in Figure 1.

Transcutaneous Electrical Nerve Stimulator (TENS-200A; Suzhou Medical Appliance Co. Ltd.) as taVNS on right ear was performed twice a day (morning and afternoon) for 30 min, 5 times a week. The stimulation parameters included the following: (1) a dilatational wave of 20/4 Hz (20 Hz for 7 s, 4 Hz for 3 s, the sparse and dense wave alternated). (2) intensity set to patient tolerance with no pain. We asked the patient’s family to record of his everyday situation, and conducted telephone interviews with both the patient and his family to promptly report any adverse reactions.

### DTI data acquisition and analysis

All DTI data were collected using a 3-Tesla MRI scanner (SIEMENS VERIO, Erlangen, Germany) with an 8-channel head coil at Yueyang Hospital of Integrated Traditional Chinese and Western Medicine, Shanghai University of Chinese Traditional Medicine, China. The patient had 4 scans total at t0, t1, t2 and t3. Each scan session included DTI and T1-weighted structural scans. The acquisition parameters were used for T1-weighted structural scan as follows: repetition time (TR) = 1900 ms; echo time (TE) = 2.93 ms; field of view (FOV) = 256 mm × 256 mm; flip angle = 9°; acquisition matrix size = 256 × 256; voxel size = 1 mm × 1 mm × 1 mm; number of slices = 160; slice thickness = 1 mm, with no gap. The acquisition parameters were used for DTI scan as follows: TR = 10,000 ms; TE = 89 ms; FOV = 240 mm × 240 mm; flip angle = 90°; acquisition matrix size = 128 × 128; direction = 62; b = 0, 1,000 s/mm^2^; slice thickness = 2 mm and slice gap = 0.

All DTI data were preprocessed using FSL software^1^ in order to obtain FA maps of the patient each data collection time point (Menke et al., 2014). Then, Diffusion Toolkit^2^ was used to track whole-brain fiber tracts. The maximum turning angle of the fiber >45° or the FA < 0.2 was set as the terminating conditions. To extract individual region of interest (ROI) FA, the FA maps were warped to the T1-weighted structural image in the native diffusion space using affine registration (FLIRT) and non-linear registration (FNIRT) registration algorithms (Menke et al., 2014). The automated anatomical labeling (AAL) atlas^3^ was warped from the MNI space to the native diffusion space (Qiu et al., 2017). We used the bilateral DLPFC (left DLPFC: Frontal_Sup_L and Frontal_Sup_Medial_L; right DLPFC: Frontal_Sup_R and Frontal_Sup_Medial_R), the bilateral ACC (Cingulum_Ant_L and Cingulum_Ant_R), the bilateral PCC (Cingulum_Post_L and Cingulum_Post_R), the bilateral hippocampus (Hippocampus_L and Hippocampus_R) and the bilateral thalamus (Thalamus_L and Thalamus_R) as the ROIs. Finally, the mean value of FA was extracted from each ROI.

## Results

### Behavior data

**Figure 2:**
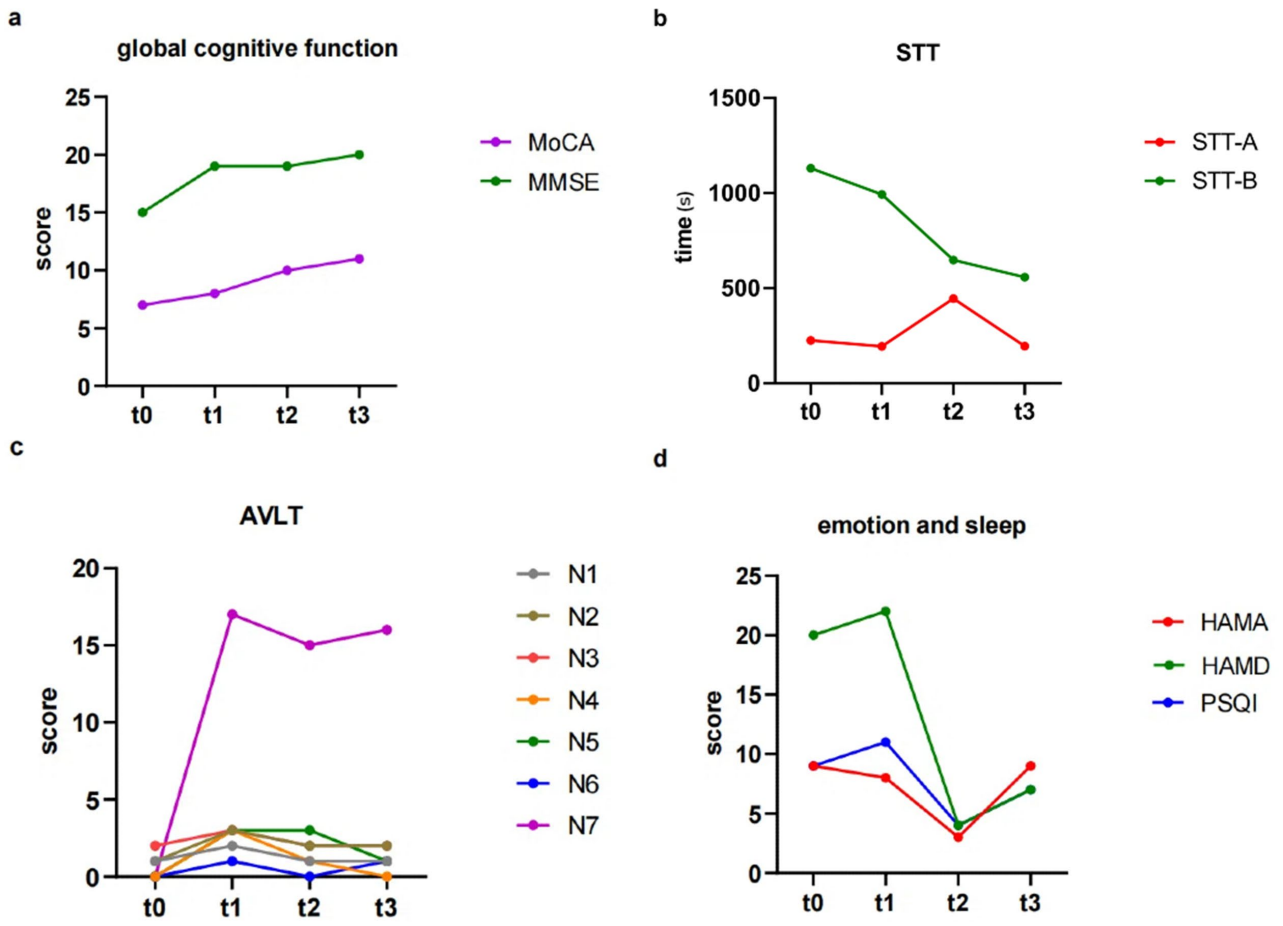
The results of behavior data. **(a)** Changes in global cognitive function. **(b)** Changes in STT. **(c)** Changes in AVLT. **(d)** Changes in emotion and sleep. STT, shape trails test; AVLT, auditory verbal learning test-HuaShan version.

The patient’s family reported no discomfort during the taVNS treatment, and the patient’s habits and diet remained consistent. At baseline (t0), the patient’s MoCA score was 7. Following four weeks of living without any intervention (t1), the score was 8. After 4 weeks of intervention (t2) the score increased to 10, and further increased to 11 after eight weeks of intervention (t3) (Table 1; Figure 2a).

**Table 1:** The behavior data scores of the patient (t0, t1, t2, and t3).

	Behavior data scores			
	t0	t1	t2	t3
MMSE	15	19	19	20
MoCA	7	8	10	11
AVLT				
N1	1	2	1	1
N2	1	3	2	2
N3	2	3	2	2
N4	0	3	1	0
N5	0	3	3	1
N6	0	1	0	1
Recognition	0	17	15	16
STT-A (s)	225.63	194.84	445.00	195.08
STT-B (s)	1130.00	992.75	648.00	557.86
HAMA	9	8	3	9
HAMD	20	22	4	7
PSQI	9	11	4	7

t0, baseline; t1, 4 weeks after baseline without intervention; t2, 4 weeks after intervention; t3, 8 weeks after intervention. MMSE, mini-mental state examination; MoCA, Montreal cognitive assessment; AVLT, auditory verbal learning test-HuaShan version; STT, shape trails test; HAMA, Hamilton anxiety scale HAMD, Hamilton depression scale; PSQI, Pittsburgh sleep quality index.

In contrast, the MMSE showed no significant trend during the trial period. The scores at t0, t1, t2, t3 were 15, 19, 19, and 20, respectively (Table 1; Figure 2a).

Regarding STT, STT-A, assessing attention, showed no significant change in the time spent at each time point (t0, t1, t2, t3) with values of 225.63, 194.84, 445.00, 195.08 [seconds], respectively. On the other hand, STT-B, assessing executive function, showed a notable improvement during the intervention period, with patient requiring less time to complete the test at the four time points (1130.00, 992.75, 648.00, and 557.86 s), compared to when there was no taVNS intervention (Table 1; Figure 2b). Regarding AVLT, immediate recall abilities (N1, N2, and N3) showed no significant changes over the four time points. The fractions of N1 are 1, 2, 1, and 1; for N2, 1, 3, 2, and 2; and for N3, 2, 3, 2, and 2. N5, which assessed long delayed recall ability, with scores of 0, 3, 3, and 1 at each of the four time points, and scores of 0, 17, 15, and 16 for the recognition component at each time point, all of which showed no significant change (Table 1; Figure 2c).

During the period between t2 and t3, the patient and his family had a violent quarrel due to financial reasons, and the family reported that the patient seemed to be in a bad mood and low motivation since the argument. Emotionally, the HAMA showed that the subject showed improvement in anxiety symptoms during the first 4 weeks of taVNS treatment, with scores dropping from 8 to 3. However, there was a recurrence of anxiety symptoms in the latter 4 weeks (with HAMA scores rising to 9 at t3). The HAMD results showed scores of 20 and 22 at t0 and t1, respectively; after receiving the intervention, scores were 4 and 7 at t2 and t3, separately. Patients’ depressive symptoms improved after receiving taVNS compared to the two phases (Table 1; Figure 2d).

In terms of sleep, the PSQI showed scores of 9 and 11 in the absence of intervention (t0 and t1), which then decreased to 4 after 4 weeks of taVNS, indicating an improvement of patients’ sleep quality. However, in the latter 4 weeks of intervention PSQI scores increased to 7, suggesting a decrease in sleep quality. Table 1 showed the behavior data scores of the patient at each time point.

### DTI data

**Figure 3:**
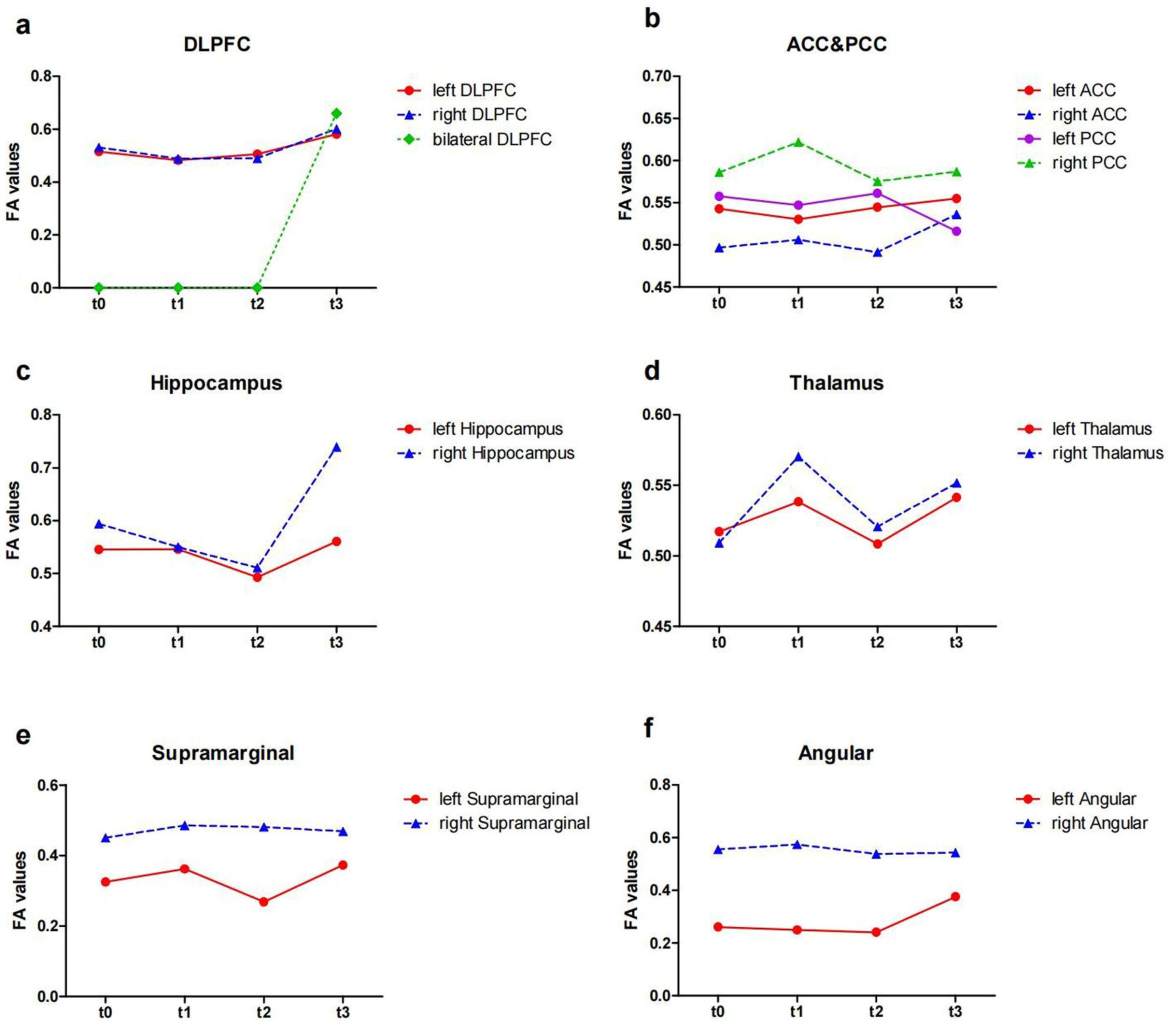
The FA values from ROIs of the patient (t0, t1, t2 and t3). **(a)** The FA values in the DLPFC; **(b)** the FA values in the ACC and PCC; **(c)** the FA values in the Hippocampus; **(d)** the FA values in the Thalamus; **(e)** the FA values in the Supramarginal; **(f)** the FA values in the Angular. FA, fractional anisotropy; ROI, region of interest; DLPFC, dorsolateral prefrontal cortex; ACC, anterior cingulate cortex; PCC, posterior cingulate cortex; t0, baseline; t1, 4 weeks after baseline without intervention; t2, 4 weeks after intervention; t3, 8 weeks after intervention.

**Figure 4:**
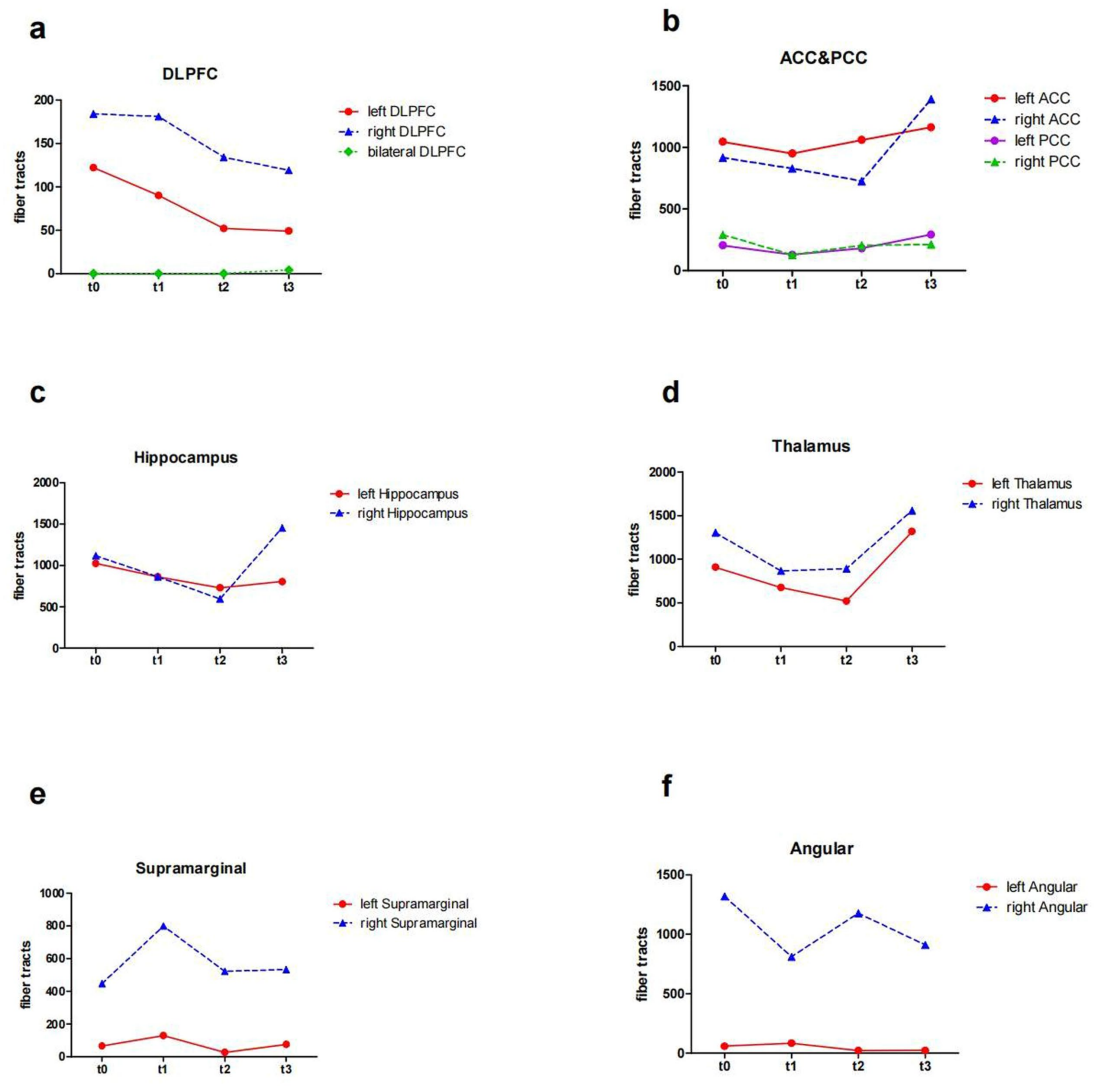
The number of fiber tracts from ROIs of the patient (t0, t1, t2 and t3). **(a)** the number of fiber tracts in the DLPFC; **(b)** the number of fiber tracts in the ACC and PCC; **(c)** the number of fiber tracts in the Hippocampus; **(d)** the number of fiber tracts in the Thalamus; **(e)** the number of fiber tracts in the SupraMarginal; **(f)** the number of fiber tracts in the Angular. ROI, region of interest; DLPFC, dorsolateral prefrontal cortex; ACC, anterior cingulate cortex; PCC, posterior cingulate cortex; t0, baseline; t1, 4 weeks after baseline without intervention; t2, 4 weeks after intervention; t3, 8 weeks after intervention.

At baseline, before the initiation of the treatment (t0 and t1), the results showed reduced FA values in the left DLPFC, right DLPFC and left ACC (Table 2; Figure 3). After the treatment (t2 and t3), the FA values in these ROIs were subsequently increased (Table 2; Figure 3). Similarly, the decreased number of fiber tracts in the left ACC, left PCC, right PCC and right thalamus observed prior to the treatment increased post the treatment (Table 3; Figure 4). These results were consistent with the behavioral results.

**Table 2:** The FA values from ROIs of the patient (t0, t1, t2, and t3).

	FA values (mean ± SD)			
	t0	t1	t2	t3
Left DLPFC	0.515546 ± 0.126256	0.482298 ± 0.158521	0.505693 ± 0.154693	0.581205 ± 0.164973
Right DLPFC	0.530893 ± 0.154891	0.488349 ± 0.144625	0.489671 ± 0.133767	0.601236 ± 0.160932
Bilateral DLPFC	0	0	0	0.660146 ± 0.153723
Left ACC	0.542786 ± 0.161377	0.530512 ± 0.159607	0.544636 ± 0.156398	0.555200 ± 0.155973
Right ACC	0.496703 ± 0.146860	0.506063 ± 0.156758	0.491279 ± 0.146987	0.536186 ± 0.153576
Left PCC	0.557581 ± 0.154165	0.547122 ± 0.179424	0.561405 ± 0.177470	0.516437 ± 0.157602
Right PCC	0.585838 ± 0.118165	0.621691 ± 0.187571	0.575283 ± 0.128870	0.586830 ± 0.148985
Left Hippocampus	0.545657 ± 0.144594	0.545813 ± 0.171751	0.492800 ± 0.142462	0.560647 ± 0.158447
Right Hippocampus	0.593796 ± 0.181242	0.550097 ± 0.168247	0.510966 ± 0.163347	0.739227 ± 0.177355
Left Thalamus	0.517213 ± 0.156864	0.538497 ± 0.175191	0.508432 ± 0.155435	0.541396 ± 0.179948
Right Thalamus	0.509219 ± 0.150010	0.570285 ± 0.172985	0.520737 ± 0.160423	0.551718 ± 0.162511
Left Supramarginal	0.324865 ± 0.103233	0.362453 ± 0.112139	0.268231 ± 0.065067	0.372718 ± 0.113100
Right Supramarginal	0.450374 ± 0.127879	0.485388 ± 0.134930	0.480543 ± 0.140268	0.468455 ± 0.128942
Left Angular	0.261181 ± 0.057556	0.249850 ± 0.037677	0.241302 ± 0.056893	0.375398 ± 0.149067
Right Angular	0.554801 ± 0.163894	0.573882 ± 0.163452	0.537636 ± 0.143805	0.542962 ± 0.149138

FA, fractional anisotropy; ROI, region of interest; DLPFC, dorsolateral prefrontal cortex; ACC, anterior cingulate cortex; PCC, posterior cingulate cortex; t0, baseline; t1, 4 weeks after baseline without intervention; t2, 4 weeks after intervention; t3, 8 weeks after intervention.

**Table 3:** The number of fiber tracts from ROIs of the patient (t0, t1, t2, and t3).

	Number of fiber tracts			
	t0	t1	t2	t3
Left DLPFC	122	90	52	49
Right DLPFC	184	181	134	119
Bilateral DLPFC	0	0	0	4
Left ACC	1,045	951	1,060	1,163
Right ACC	917	828	725	1,391
Left PCC	204	128	181	292
Right PCC	290	127	204	212
Left Hippocampus	1,024	864	729	806
Right Hippocampus	1,118	863	595	1,454
Left Thalamus	909	678	523	1,319
Right Thalamus	1,304	867	892	1,558
Left Supramarginal	65	129	26	75
Right Supramarginal	447	799	522	533
Left Angular	58	83	20	22
Right Angular	1,319	810	1,177	911

ROI, region of interest; DLPFC, dorsolateral prefrontal cortex; ACC, anterior cingulate cortex; PCC, posterior cingulate cortex; t0, baseline; t1, 4 weeks after baseline without intervention; t2, 4 weeks after intervention; t3, 8 weeks after intervention.

In addition, the number of white matter fiber tracts continued to decrease in the left DLPFC and right DLPFC, either before or after the treatment (Table 3; Figure 4a). However, after 8 weeks of treatment (t3), the fiber tracts passing through the bilateral DLPFC increased significantly (Table 3; Figure 4a).

## Discussion

It has been shown that taVNS has an ameliorative effect on cognitive function in patients with mild cognitive impairment and healthy individuals, in terms of global cognitive function, memory function, or executive function (Ridgewell et al., 2021; Wang et al., 2022). This provides the basis for taVNS being a possible treatment for PSCI. Our case focused on long-term PSCI patient who was treated with taVNS for 8 weeks. We analyzed the white matter remodeling effects of taVNS on long-term PSCI by DTI to observe the feasibility of taVNS as a home-based treatment for long-term PSCI patient.

The ease of use of the machine was praised by the patient’s family, who said that the patient did not experience discomfort during the treatment and that the patient was proactive throughout the treatment cycle. Our results showed that 8 weeks of taVNS can improve MoCA, STT-B to varying degree, suggesting that taVNS improved global cognitive function and executive function in this long-term PSCI patient. The completion of STT-B is more dependent on executive function and memory, and its shortening completion time may indicate the improvement of function (Zhao et al., 2013). Meanwhile, the patient’s mood and sleep symptoms fluctuated during taVNS treatment despite improvement, possibly related to family troubles that occurred during the period from T2 to T3. It has been shown that emotional symptoms and sleep can cause cognitive decline (Koppel et al., 2012; Lo et al., 2014). We therefore hypothesized that the changes in mood and sleep problems observed during the period between t2 and t3, may influence the improvement of cognitive function by taVNS.

Our DTI results showed the FA values that declined in left right DLPFC before treatment, increased after taVNS. Furthermore, disorders of white matter integrity in the angular and supramarginal gyrus were adjusted during treatment. FA value can measure the integrity of white matter microstructure, reflecting potential changes in axon diameter, density, and myelination (Lim et al., 2015). The DLPFC plays an important role in cognitive function such as memory (Barbey et al., 2013a,b), executive function (Dubreuil-Vall et al., 2019), and attention (Kang et al., 2009), and is involved in regulating emotions (Salehinejad et al., 2017). It has been shown that FA values in frontal regions correlate with executive function (Mayo et al., 2018). The supramarginal gyrus and angular gyrus constitute the posterior parietal cortex (PPC). The PPC and DLPFC are coactivated in cognitive control during cognitive operations that requiring attention and working memory, but inactivation of the PFC leads to more severe impairments of coginitive function such as memory, executive function, and attention, and is involved in regulating emotions in these cognitive domains (Katsuki and Constantinidis, 2012).

In our DTI results, the number of fiber tracts in the bilateral DLPFC continued to decrease after 8-week taVNS, while the number of the fiber tracts passing through the bilateral DLPFC increased after 8-week taVNS treatment. Atrophy of the corpus callosum consisting of interhemispheric fiber tracts, may be an important predictor of global cognitive impairment, and slowing damage to these interhemispheric fiber tracts may prevent the development of dementia (Yamauchim, 2003). Patients in the early stage of stroke may exhibit only intraregional white matter damage, whereas the exacerbation of vascular risk factors may lead to progression of white matter damage to concomitant corpus callosum atrophy, affecting interhemispheric connectivity and cooperation. Increased fiber tracts between hemispheres and enhanced white matter tracts integrity can increase information transfer (Yang et al., 2022). The fiber tracts within the unilateral DLPFC have some short connections, whereas the fiber tracts between bilateral DLPFC are long connections. The reorganization of both short and long connections within and between the fiber tracts of the DLPFC, along with changes of white matter integrity in the DLPFC and PPC, improves the efficiency of information conduction. This provides a structural basis for the functional reorganization of the central nervous system.

In cognitive control system, DLPFC provides top-down support for task-appropriate behaviors, while the ACC may be involved in the processes of assessment and monitoring that indicate when more robust control is needed (Badre and Wagner, 2004; MacDonald et al., 2000). Our results showed a rise in FA in the left ACC after taVNS, which were consistent with the behavioral results. A study demonstrated that FA in the left ACC was associated with strategic game learning (Ray et al., 2017). This may also be the reason behind the patient’s improved performance in STT-B, who successfully choose the correct answer among the interfering items. But the patient forgot the number when he went through the cueing errors during the STT-B test and did not know how to proceed. It has been shown that the PCC supports change monitoring, engages in strategy control and switching (Pearson et al., 2011). In our results, the FA values of the PCC did not change much, which could explain this patient’s inability to cope with sudden changes in events. Nevertheless, we found an increase in the number of the PCC fiber tracts in both hemispheres, which increased information transfer (Yang et al., 2022), perhaps indicating a positive phenomenon. This system, in which the ACC and DLPFC are located, is also associated with emotions, and changes in the white matter microstructure within this system can also alleviate emotional symptoms.

It has been shown that patients with Alzheimer’s disease have reduced FA values in the hippocampus (Fellgiebel et al., 2004). In patients with mild cognitive impairment, reduced hippocampus-related fiber tracts were associated with cognitive dysfunction; reduced thalamus-related fiber tracts were associated with depressive symptoms (Zhou et al., 2022). The thalamus is a key integrating center within the brain’s functional networks, maintaining functional and structural connection with multiple cortical networks. Through information transfer and convergence with these networks, the thalamus can facilitate a variety of cognitive functions (Hwang et al., 2017). Structural and functional alterations in the thalamus influence the severity of cognitive impairment (Schoonheim et al., 2015). Study showed that a mouse model of thalamic lacunar infarction exhibited impaired learning and memory (Zhang et al., 2023).

We found that after taVNS, the fiber tracts in the hippocampus and thalamus of this patient fluctuated during treatment, but there was an elevation after 8 weeks, compared to the untreated period. The modulation of white matter integrity disturbances within the hippocampus and thalamus by taVNS also provides a basis for improved cognitive functioning.

Although this case report demonstrates the potential of home-based taVNS to affect the brain structurally and functionally in patients with long-term PSCI, the fact that there was only one patient makes the results limited in terms of generalization. Furthermore, this case report lacks a follow-up, leaving us uncertain about the duration of the taVNS effect. Therefore, randomized controlled trials with larger sample sizes are still needed to explore the effects of home-based taVNS on the progression of cognitive impairment in patients with long-term PSCI.

## Conclusion

The findings presented in this case report provide a potentially viable taVNS treatment option for home-bound patients with long-term PSCI. The behavioral and DTI results recorded also show the potential benefits of this approach.

## Data Availability

The original contributions presented in the study are included in the article/Supplementary material, further inquiries can be directed to the corresponding author.

## Footnotes

^1^ http://www.fmrib.ox.ac.uk/fsl

^2^ http://www.trackvis.org/dtk/

^3^ http://www.gin.cnrs.fr/AAL
